# Repeated Sprint Training in Hypoxia: Case Report of Performance Benefits in a Professional Cyclist

**DOI:** 10.3389/fspor.2020.00035

**Published:** 2020-04-14

**Authors:** Raphael Faiss, Arnaud Rapillard

**Affiliations:** ^1^Research and Expertise in anti-Doping sciences (REDs), Institute of Sport Sciences, University of Lausanne, Lausanne, Switzerland; ^2^Clinique romande de réadaptation, SUVACare, Sport Medicine Unit, Sion, Switzerland

**Keywords:** hypoxia, cycling, sprint, performance, case study

## Abstract

Repeated sprint training in hypoxia (RSH) has gained unprecedented popularity among the various strategies using hypoxia as an additional stimulus to improve performance. This case study reports the benefits of 150 repeated sprints in normobaric hypoxia over 10 days in a professional cyclist. After 3 weeks of endurance training in November, the cyclist performed five RSH sessions at a simulated altitude of 3,300 m on his own bicycle attached to an indoor trainer in a hypoxic chamber (FiO_2_ 14.1 ± 0.1%, PiO_2_ 94.6 ± 1.4 mm Hg). Each session consisted of four blocks of seven all-out sprints of 6 s interspersed with 14 s active recovery (for a total of 126 s per block). After 12 min of warm-up with a single isolated 6 s reference sprint, the sessions included a first and a second sprinting block with 4 min 54 s active recovery in-between. After 9 min 54 s active recovery including an isolated 6 s reference sprint, a third and a fourth block were performed with 4 min 54 s active recovery in-between, before an active cool-down of 9 min 54 s. The total duration was thus of 50 min *per session* for a total hypoxic exposure of 250 min exercising. Power output and heart rate were monitored at 1 Hz. Lactate concentration ([La]) and pulse oxygen saturation (SpO_2_) were measured at the start and end of each block during the first and fifth training session. Basal SpO_2_ was of 83% during session one and 85.5% during session five. When comparing the first and fifth training session, peak power increased for the best 1 s value (+8%) and the best 5 s average (+10%) to reach 1,041 W and 961 W, respectively. Average power for all blocks (including active recoveries) increased from 334 to 354 W with a similar average heart rate during the sessions (146'^.^min^−1^). Peak [La] was increased from 12.3 to 13.8 mmol^.^l^−1^. In conclusion, this case report illustrates a 10-days RSH intervention perceived as efficient in a professional cyclist and shown to improve total work (6-s sprints) produced for a similar physiological strain.

## Introduction

Many athletes feel attracted to include altitude training interventions in their preparation anticipating additional performance gains in comparison to equivalent sea-level training. However, such benefits for elite athletes are still sharply debated (Millet and Brocherie, 2020).

In that context, the repeated sprint training in hypoxia (RSH) strategy was subject to an unprecedented interest with 25 studies published in the last 7 years (Millet et al., 2019) after the seminal study showing significant performance gains and strong adaptations at the muscular level in cyclists (Faiss et al., 2013b). The RSH was designed with an additional hypoxic stress to promote peripheral adaptations combined with a maximal recruitment of fast twitch fibers with a superior oxygen (O_2_) extraction (when compared to their slow twitch counterparts) (Mcdonough et al., 2005) during the intensity-dependent hypoxic compensatory vasodilation (Casey and Joyner, 2012). For instance, almost all studies reporting benefits of RSH compared to the same training in normoxia (Millet et al., 2019) and a recent meta-analysis calculated the additional gain to be ~2% for repeated sprinting efforts (Brocherie et al., 2017). Interestingly, several studies have reported such benefits also in highly competitive endurance and team-sport athletes with striking evidence that the (little) gains may be obtained even after a very short training block (Faiss et al., 2013a, 2014; Beard et al., 2019). The latter is particularly important for elite athletes with a very tight training agenda and often only little flexibility to include training blocks in a single location if lasting more than 2 weeks. While cycling is essentially aerobic with efforts lasting up to several hours (Faria et al., 2005), modern bicycle road racing includes however very stochastic high-intensity bouts with multiple rapid decelerations and accelerations (Menaspà et al., 2017). The ability to “sprint after having sprinted” was hence recently tested with greater reduction in a “final” 30 s sprint after a 1 h cycling trial if the latter includes multiple high-intensity bouts (Etxebarria et al., 2019). These authors indeed highlight the need to improve repeated sprint ability to optimize a final sprint and hence maintain a high performance level in competitive cycling.

In the current panorama of hypoxic training strategies, RSH may thus represent an interesting option among the various “Live Low—Train High” possibilities (Girard et al., 2017) while there is no firm recommendation or practical example on how RSH should be implemented in elite athletes. Personal communications with athletes and trainers have however allowed our group to test various options (i.e., regarding number and timing of sessions, exercise to rest ratio, and sprint duration/number) to propose attractive RSH strategies with good results and positive subjective feedbacks from professional athletes across several endurance and team sports.

The aim of this case report is thus to present the effects on performance of a RSH block of five training sessions within 10 days in a professional cyclist.

## Case Description

This report showcases a descriptive study of a professional athlete competing at the Union Cycliste International (UCI) Continental and World Tour levels beginning his winter preparation with a block of RSH training after a 4-weeks break without cycling in October and 3 weeks of endurance training in November. The cyclist is 27 years old (69 kg, 179 cm) and boasts a 7 years career as an elite cyclist out of which three riding in UCI World Tour races and four at the UCI Continental level. Over the last training season, his average training volume reached 18.8 h of cycling weekly for a total distance >22,000 km (out of which >9,000 km over >60 race days). The subject was not sick nor exposed to an altitude >1,500 m for the 3 weeks preceding the training intervention. It is important to mention that he was completing a RSH training block for the fifth time and therefore benefited from some experience on the expected feelings or outcomes for the intervention. No other RSH block was however performed in the last 10 months preceding this intervention. The subject provided an informed written consent to participate in the training intervention and the study was conducted in respect of the Declaration of Helsinki.

## Training Intervention and Measured Variables

At the end of the season, the athlete spent 26 days without training before including endurance training cycling sessions progressively starting the first week of November for a total of 43 training hours before the RSH intervention. The RSH intervention was planned in a 14-weeks training plan before the first stage race of the 2020 season (Vuelta a San Juan Internacional) at the end of January. The athlete was asked to produce a maximal 6 s sprint on his road bike during a training ride 1 week before the intervention to serve as a reference. The RSH block lasted for 10 days and the cyclist reported to the lab in the morning to perform five RSH sessions followed immediately by an endurance recovery road ride of ~90 min. RSH sessions started on a Wednesday and were then scheduled on the following Friday, Monday, Wednesday, and Friday. This design allowed for endurance rides when there was only one recovery day between RSH sessions and for two longer low-intensity road rides (for a total of 360 min) after the second RSH session. During the day preceding the first and last training session, a 120 min easy (i.e., 200 W average power) ride was scheduled.

**Figure 1:**
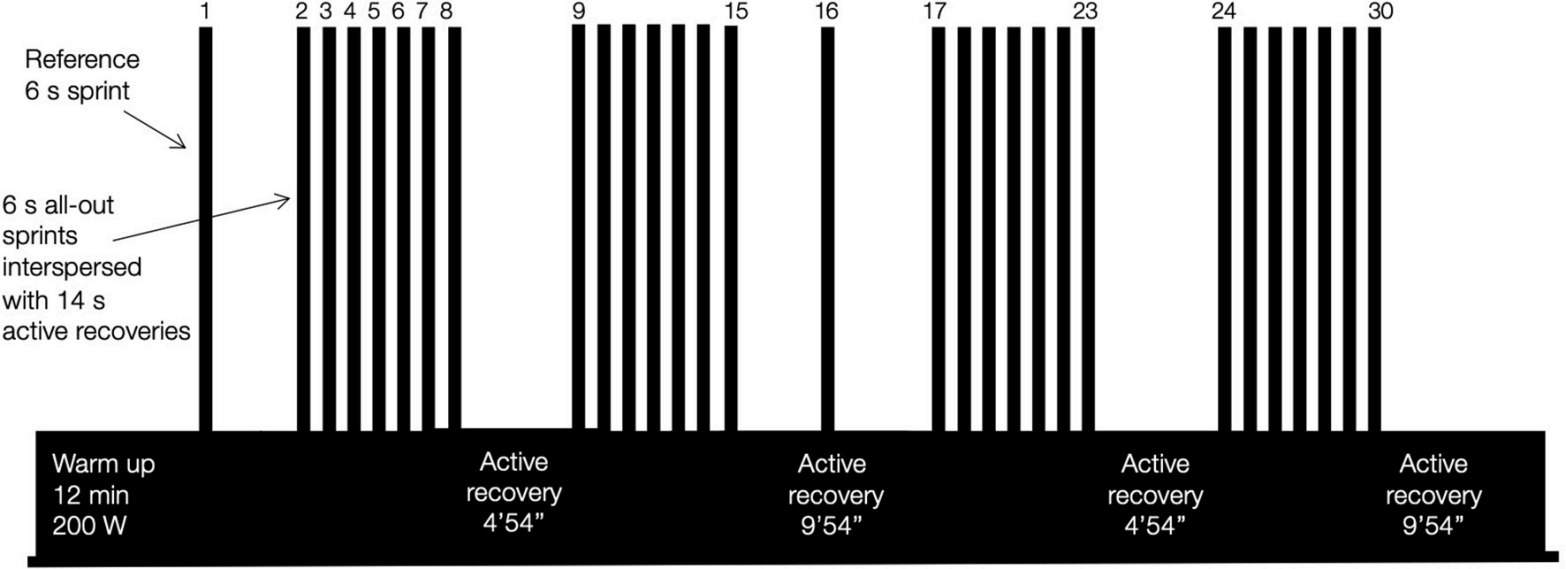
Illustration of one repeated sprint training in hypoxia session performed at a simulated altitude of 3,300 m including 30 sprints of 6 s interspersed with 14 s of active recovery.

Each training session consisted in two isolated reference sprints and four blocks of 7 all-out sprints lasting 6 s interspersed by 14 s of active recovery [targeting 200 W for optimal lactate removal/pyruvate reythesis (Brooks, 2020)]. In detail, after 12 min of warm-up with a single isolated 6 s reference sprint, the sessions included a first and a second sprinting block with 4 min 54 s active recovery in-between. After 9 min 54 s active recovery including an isolated 6 s reference sprint, a third and a fourth block were performed with 4 min 54 s active recovery in-between, before an active cool-down of 9 min 54 s. Figure 1 illustrates the design of one training session.

Each RSH session lasted 50 min in a normobaric hypoxic chamber (17 m^2^, ~50 m^3^ ATS Altitude, Sydney, Australia) located at an altitude of 500 m simulating an altitude of 3,300 m by lowering the inspired oxygen fraction (FiO_2_) to ~14 % (depending on the barometric pressure) to obtain a stable inspired oxygen pressure (PiO_2_) of 96 mmHg. Barometric pressure (BaP) and FiO2 were measured before each training session with a precise electronic oximeter (GOX 100, Greisinger, Regenstauf, Germany) and barometer (GPB 2300, Greisinger, Regenstauf, Germany), respectively. Despite slight variations in barometric pressure due to weather changes, the resulting PiO_2_ [calculated as FiO_2_ x (BaP−47)] was very stable at 94.6 ± 1.4 mmHg for a corresponding simulated altitude of 3,300 ± 61 m. Temperature was kept stable close to 24°C and a fan was used to circulate the air in the chamber.

Training sessions were performed on the cyclists own bicycle attached to an indoor trainer (Cycleops Jetfluid Pro, Saris, Madison, USA) with a hydraulic resistance providing a progressive power curve from the resistance unit (Cycleops, 2018). The cyclist was allowed to drink only water *ad libitum* and a fan was used to facilitate convective cooling during sessions.

Power output was measured with a crank-based power meter (Stages Gen 3, Stages Power, Boulder, USA) mounted on the bicycle. The power meter was factory-calibrated one month before the intervention. Heart rate was measured with a dedicated chest strap (Garmin HRM Dual, Olathe, USA). Power output and heart rate were recorded throughout the training sessions at 1 Hz by telemetry on a bicycle computer (Garmin Edge 530, Olathe, USA). Training load was calculated from the training volume (in h) and with a dedicated compound score [i.e., Training Stress Score (TSS®)]. Briefly, the TSS is a way to express workload numerically from a single training session with the following formula TSS (a.u.) = (duration (s) x Intensity factor x Normalized power)/(Functional threshold power × 3,600) × 100; where the functional threshold power (FTP) represent the highest average power maintained during 60 min (in a previous training session or race), Intensity factor represent the fraction of the FTP of the Normalized power (NP) of the entire session. In turn, NP is an estimate of the power that could have been maintained at a constant pace and it is calculated by taking the fourth root of the mean values of 30-s rolling averages (of the original power output data recorded) elevated to the fourth power (Allen and Coggan, 2010).

Lactate concentration ([La]) was measured during the first and last training session at rest before warming up and in the 30 s following each sprinting block with a hand-held analyser (Lactate Pro, Nova Biomedical, Waltham, USA). Pulse oxygen saturation (SpO_2_) was measured with a wrist worn oximeter (Pulox PO-400, Contech Medical Systems, Qinhuangdao, China) during the first and last training session to assess basal SpO2 (defined as defined as average of all SpO2 readings not included in a desaturation event) during warm-up and the lowest saturation measured during the session. Rate of perceived exertion (RPE) was recorded after each session with a 1–10 Borg scale. Data are presented as means (SD).

## Outcomes and Results

**Figure 2:**
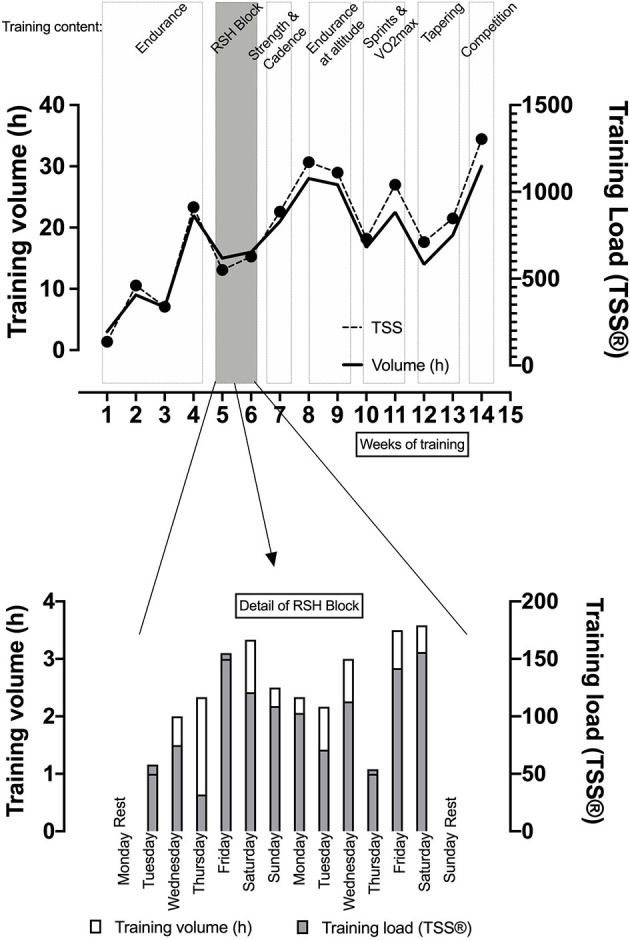
Description of the 10-days training intervention as part of a 14-weeks training plan with description of general training content including training volume (in hours) and training load calculated as a Training Stress Score® [TSS, arbitrary unit, with the formula of Allen and Coggan, 2010]. RSH, Repeated Sprint training in Hypoxia.

During the 10 days intervention, the cyclist completed 1,500 min of training out of which 250 were done in the hypoxic chamber for the RSH sessions. Overall, during the 14 weeks training period considered including the RSH intervention, the cyclist completed a total volume of 250 training hours and a cumulated TSS of 10826 (a.u.). Figure 2 illustrates the training volume and training load for the 14 weeks of the training period including the detail of the 10 days of the RSH intervention.

The cyclist reached a reference 5 s peak power of 1,169 W during a road cycling training ride 6 days before the intervention.

The good adherence to the training program and willingness to perform all-out sprints without pacing was confirmed by the consistent difference between the highest and lowest 1 s peak power attained during all blocks averaging −15.0 ± 2.4% over all training sessions. RPE was ranging between 8 (3rd session) and 10 with an average of 9 ± 0.7. There were no advert or unanticipated events occurring during the training intervention.

Peak 1 s power from all five sessions was in average 896 ± 33 during the first blocks, 906 ± 39 W for the second blocks, 897 ± 45 W during the third blocks and 847 ± 44 W for the fourth blocks.

Table 1 describes the peak and average power output recorded during isolated reference sprints (1 s peak, overall) and the successive repeated sprint blocks (1 and 5 s peaks) for sessions one and five. The average power during the sprinting blocks was of 342 ± 11 W over the five session (ranging from 334 during session one to 354 during session five).

**Table 1:** Power output during isolated and repeated sprints.

	**Peak 1 s (W)**					**Peak 5 s (W)**	**Average (W)**
	**Overall**	**Block 1**	**Block 2**	**Block3**	**Block 4**	**All blocks**	**All blocks**
Session 1	961	866	961	906	850	867	334
Session 2	1,004	932	890	956	909	888	336
Session 3	1,039	900	860	884	788	923	344
Session 4	976	924	890	832	858	916	348
Session 5	1,041	858	928	907	829	961	354
Difference	+8%	−1%	−3%	–	−2%	+11%	+6%

The peak 1 s power recorded during the isolated sprint was systematically higher during the second isolated sprint (i.e., after two blocks of repeated sprints) with 998 ± 47 W vs. 932 ± 9 W, respectively. The highest 1 s power was attained during the second isolated sprint during session five (1,041 W, Table 1).

The best 5 s peak (representing the best power output for a full 6 s sprint) improved by 11% between session one and session five. Similarly, the total work produced during sprinting blocks increased by 6% (as illustrated by the average power of all blocks in Table 1).

The average power and heart rate for the whole session during the five hypoxic training sessions were of 199 ± 5 W and 150 ± 6 min^−1^. Table 2 describes the physiological responses observed during the first and last training sessions.

**Table 2:** Physiological responses to the first and last repeated sprint training sessions.

	**Lactate concentration (mmol**^**.**^**l**^****−1****^**)**					**Heart Rate (‘**^**.**^**min**^****−1****^**)**		**Basal SpO_**2**_**	**Lowest SpO_**2**_**
	**Rest**	**Block 1**	**Block 2**	**Block3**	**Block 4**	**Average**	**Peak**	**Warm-up**	**All session**
Session 1	1.8	8.7	11.8	11.8	12.3	146	180	83.5%	76%
Session 5	1.3	9	13	12.9	13.8	146	177	85%	76%
Difference	−27%	+3%	+10%	+9%	+12%		−2%	+2%	

## Discussion

The present case study illustrates the use of a normobaric hypoxic chamber to provide an additional hypoxic stress during five sessions of repeated sprints in a professional cyclist. The main outcome is a 6% improvement of the total work produced during sprinting blocks at the fifth session for a similar physiological strain (same average heart rate). The best sprint power (over 5 s) was improved by 11% over the 10 days intervention. Interestingly, peak 1 s power was not higher during the fifth training session. However, with a higher average power attained during the sprinting block, it can be speculated that fatigue could have been delayed during sprinting with better maintained 5 s power and slower decrement of power when sprints are repeated.

Our results indicate a strong additional stimulus of the hypoxic environment resulting in moderate hypoxia with the occurrence of some very low levels of oxygen saturation (e.g., 76%) during sprinting. The aim of such training protocol was to stimulate the glycolytic energetic pathway and to improve oxygen delivery and extraction at the peripheral level. The latter is supported by indicators of an enhanced muscle perfusion during RSH efforts from earlier studies (Faiss et al., 2013b, 2014). The rationale of proposing short 6 s sprints is to maximize the use of fast twitch fibers with a higher O2 extraction potential (Mcdonough et al., 2005). The design of the session and the work:rest ratio proposed may be particularly favorable for the concept of intensity-dependant compensatory vasodilation to match oxygen delivery to the demand during such hypoxic exercise (Casey and Joyner, 2012). In particular, the nitric oxide (NO) mediated vasodilation [especially marked in fast twitch fibers utilized when sprinting (Copp et al., 2013)] may be repeatedly stimulated and could explain why the cyclist was able to produce more power during sprinting blocks after five sessions with the same average heart rate. Hypoxia is undoubtedly a potent stimulus challenging oxygen homeostasis through the hypoxia-inducible factor family (HIFs). For instance, adaptations observed at the molecular level after RSH suggest a HIF-regulated transcriptional response of genes involved in the muscle pH regulation (Faiss et al., 2013b). The latter supports a shift toward an enhanced glycolysis that may be supported by the increased [La] observed after the RSH intervention here. For instance, maximal [La] measured at the end of each sprinting block was up to 13% higher during the fifth training session (compared to the first). This result is hence an indicator of an increased contribution of glycolysis during the effort with a possible concomitant increase in lactic tolerance (Thomas et al., 2004; Girard et al., 2011). For instance, the higher levels of [La] observed may be explained by the increased anaerobic glycolysis during high-intensity exercise performed in acute hypoxia (Morales-Alamo et al., 2012). In the specific context of RSH, molecular adaptations (upregulated mRNA expression of lactate dehydrogenase, carbonic anhydrase 3 and monocarboxylate transporter 4) were proposed as indicators of such increased activation of the glycolytic pathway observed in our subject (Faiss et al., 2013b). The concomitant improved responsiveness of the vascular bed and the higher blood perfusion through nitric oxide (NO)-mediated vasodilation mechanisms could in turn improve the phosphocreatine (PCr) resynthesis during recovery phases to maintain energy supply for the next sprint phase (Mendez-Villanueva et al., 2012; Faiss et al., 2013a). RSH is hence not expected to improve the athletes' aerobic capacity but its effectiveness may rely on a high level of perfusion of fast twitch fibers [(owing to their greater O_2_ extraction when perfused (Mcdonough et al., 2005)] to support PCr recovery mechanisms during consecutive efforts with incomplete recovery phases (Faiss et al., 2013a).

While the first study reporting benefits of RSH was conducted on well-trained (but not elite) cyclists (Faiss et al., 2013b), numerous successive studies have tried to understand the mechanisms underlying putative performance enhancement after RSH with a few testing competitive or elite athletes [e.g., cross-country-skiers (Faiss et al., 2014), field-hockey, (Brocherie et al., 2015), lacrosse (Kasai et al., 2018), rugby union (Beard et al., 2019)]. There is a large interest for training methods with benefits not only for moderately trained subjects but also for elite or professional athletes. In that context, RSH does certainly not represent a magic method with benefits occurring in very specific conditions (e.g., delayed fatigue in repeated sprints or slightly improved power outputs) as described in a recent comprehensive review (Girard et al., 2017). Hypoxic training works under the premise that the use of an additional hypoxic stress may boost performance benefits compared to similar normoxic training. This offers an explanation for the unprecedented interest (25 studies published) of the repeated sprint training in hypoxia (Millet et al., 2019). Overall, if fatigue is delayed during repeated sprint bouts by peripheral adaptations linked to oxygen delivery, one may speculate that the reactivity of the vascular bed is improved. In other words, a delayed fatigue may also be interpreted as a succession of improved recovery phases. The latter could be useful not only for subsequent sprint training session but also in support of the development of cardio-respiratory determinants of endurance performance [e.g., oxygen uptake kinetics (Jones and Burnley, 2009)]. RSH may thus be useful in the context of oxygen delivery to the working muscle both in normoxic and hypoxic environments (Calbet and Lundby, 2009). Much alike any other kind of additional stressor (training stimulus), if the training basics (sleep, recovery) are not compromised, there is no reason why hypoxic training and RSH especially may not be included in the tool box of elite athletes, in cycling or other sports. One should however not over interpret the present results based on the sole completion of a single block of RSH by a professional cyclist and consider this case report as an original illustration of a real life training scenario with a training stimulus appreciated by the athlete.

## Subject Perspective

The perspective of the athlete is indeed particularly interesting beyond any physiological or performance improvement after the RSH intervention. In his opinion, RSH allows to challenge his body and mind with a level of difficulty (due to the systemic hypoxia) that is hard to reach in habitual road cycling training in normoxia. One important feedback was that the repeated sprinting sessions were very hard with a high level of exhaustion at the end of each session. The additional use of hypoxia could be considered an asset in the context of athletes reporting that they can hardly reach the level of exhaustion during similar repeated sprint sessions performed in normoxia (Faiss et al., 2013b). The cyclist however underlined his feeling of a rather low level of central fatigue following the sessions allowing for good endurance training sessions directly on the days following RSH sessions. The psychological benefit of performing a rather unique type of training that others may not do is quite difficult to evaluate but may support the use of such training intervention in absence of mechanisms affecting negatively performance (Millet et al., 2019) or deleterious for the athletes' immune status (Born et al., 2016). The strength of this study is certainly to present some improvements [e.g., more sprinting work produced for a given physiological (heart rate) strain] even in an athlete with a very high initial level of performance. Overall, the subject included the RSH intervention in a longer 14-weeks training plan for the first seven-stages race of his season where he confirmed a very good level of performance (with three breakaways and substantial work during all stages for his teammates in sprint preparation or lead-outs).

## Limitations

A limitation of performing repeated-sprints on an indoor trainer is certainly that it is very hard to reach a maximal power similar (i.e., above 1,200 W for a 70 kg cyclist) to that observed in the field (i.e., road cycling) conditions. Due to the exponential resistance curve it may take slightly more than 6 s to obtain the highest resistance on the hydraulically-braked trainer. The latter precludes comparisons with power output obtained in road cycling conditions. However, since power is resulting from torque and angular velocity, it can be argued that fast twitch fibers are maximally recruited during the proposed 6 s (Akima et al., 2005) even if maximal power is not reached. The rationale to propose 6 s sprints is to allow more exercise to rest transitions (i.e., 30 sprints in one single session) since it is speculated that the improved muscle perfusion after the muscular occlusion during sprinting is occurring during the incomplete rest phase in-between sprints (Faiss et al., 2013b). If the underpinning mechanisms improving muscle oxygenation are verified, such design allows to perform a high number (i.e., 150) of sprints in only five training sessions. This is particularly interesting for elite or professional athletes boasting good recovery capacities to include RSH intervention as blocks before or during the competitive season.

The accuracy of the power meter used in the current study may be questioned with regards to the observed differences. If the true power outputs are difficult to define accurately, a recent study underlined the decent precision (with a coefficient of variation of 2%) for the power meter utilized in the current study (Maier et al., 2017). This indicates a rather low day-to-day variability and allows to consider the observed differences in terms of power output with confidence.

Further, one of the main limitation is the lack of a specific measure of cycling performance beyond a simple 6-s sprint before and after the RSH block. One may question the usefulness of such RSH case report if time-trial performance or other indicators of cycling performance are not being measured. Results from a recent meta-analysis on RSH underline a minimal effect on repeated sprint performance when adding hypoxia to a repeated sprint training protocol while aerobic variables (like $\dot{V}O_{2}\max$) remain largely unaffected (Brocherie et al., 2017). It was however recently proposed to improve repeated sprint ability to optimize a final sprint and hence maintain a high performance level in competitive cycling (Etxebarria et al., 2019) and RSH may consequently be a strategy to be considered. In that context, this case report is clearly limited to an illustration or practical example on how RSH could be implemented practically in the training plan of a professional cyclist.

## Conclusion

In conclusion, this case report proposes a cycling specific RSH intervention presenting positive outcomes in terms of physiological (e.g., lower heart rate for a higher work produced during sprinting) and performance (i.e., 5-s peak power during sprinting) responses in a professional cyclist. This study may contribute to the growing body of evidence supporting RSH as an innovative and useful training approach across a variety of sports and even in elite or professional athletes. Any (even small) benefits of RSH are possibly related to the use of hypoxia as an additional stressor to support and strengthen training-related physiological adaptations linked to the repetition of all-out sprints with incomplete recovery phases.

## Data Availability Statement

The datasets generated for this study are available on request to the corresponding author.

## Ethics Statement

Ethical review and approval was not required for the study on human participants in accordance with the local legislation and institutional requirements. The patients/participants provided their written informed consent to participate in this study.

## Author Contributions

RF drafted the manuscript. AR collected the data. All authors contributed to revising the manuscript and expressed their approval of the final submitted version.

### Conflict of Interest

The authors declare that the research was conducted in the absence of any commercial or financial relationships that could be construed as a potential conflict of interest.
